# Management of polycystic ovary syndrome among Indian women using myo‐inositol and D-chiro-inositol

**DOI:** 10.6026/97320630018103

**Published:** 2022-02-28

**Authors:** Lila Vyas, Anagha Pai Raiturker, Shilpi Sud, Poonam Goyyal, Mahesh Abhyankar, Santosh Revankar, Silki Walia

**Affiliations:** 1 Vyas clinic, Jaipur, India; 2 Pairaiturkar clinic, Pune, India; 3 Safal Hospital, Nagpur, India; 4 Panchsheel Hospital, New Delhi, India; 5 USV Private Limited, Mumbai, India

**Keywords:** Women health, reproductive health, insulin sensitization, ovulation

## Abstract

Myo-Inositol and D-chiro-inositol (MI-DCI) are used in the treatment of polycystic Ovary syndrome (PCOS) due to their insulin-sensitizing actions. Therefore, it is of interest to evaluate the treatment patterns, clinical effectiveness and safety of MI-DCI combination in anagement of PCOS in Indian women. Data from 50 healthcare centers across India was collected between September 2019 and February 2020 and was used in the study. Women aged 12-45 years diagnosed with PCOS, who had received MI-DCI (550-150 mg) were included. The outcome parameters were change in weight, luteinizing hormone (LH)/follicle stimulating hormone (FSH) ratio, hirsutism, blood glucose and insulin levels, HOMA-IR, and lipid profile. A total of 283 women were included (mean age: 27.74 years; body mass index: 26.89 kg/m2); of which 197 (69.61%) reported reduction in weight after treatment with MI-DCI. The hirsutism scores considerably improved after treatment and the proportion of patients with, no hirsutism increased from 31.07% to 50.51% and moderate hirsutism reduced from 32.52% to 6.12% while, there were no patients with severe hirsutism after the treatment. There was a significant reduction in LH:FSH ratio (mean difference: 0.25 mg/dL; p=0.021), free testosterone (mean difference: 1.49; p<0.001) and dehydroepiandrosterone (mean difference: 21.49; P<0.001) levels after regular use of MI-DCI tablets. Treatment with MI-DCI resulted in significant improvement in insulin levels, HOMA-IR score, Fasting plasma glucose (FPG), post-prandial plasma glucose (PPG) and lipid profile. The therapy restored menstruation and spontaneous ovulation and significantly attenuated the LH/FSH ratio. Thus, MI-DCI (550-150 mg) has shown multidimensional benefits in improving the hormonal, glycemic, and lipid profile of women with PCOS with considerable efficacy and tolerability.

## Background:

Polycystic ovarian syndrome (PCOS) is a complex and heterogeneous endocrine disorder characterized by a constellation of symptoms and clinical features, including hyper androgenism, ovulatory dysfunction, and polycystic ovarian morphology [1]. PCOS is also associated with obesity, insulin resistance, and sub-fertility [2]. Overall, almost 65-70% of women with PCOS are affected by insulin resistance and compensatory hyper insulin-emia [2]. The latter together with beta-cell dysfunction considerably increases the risk of developing other metabolic abnormalities such as type 2 diabetes (T2D), hypertension, dyslipidemia, and cardiovascular diseases [1]. In recent years, inositol has gained much attention in the reproductive clinical practice. The therapeutic basis for the use of inositols in PCOS lies in their insulin-sensitizing potential and the beneficial metabolic effects [3]. Inositol has been classified as an "insulin-sensitizing agent" and it is mainly used as a chronic treatment for PCOS. There are two Inositol stereo isomers, myo-inositol and D-chiro-inositol (MI-DCI). Both function as insulin second messengers and mediate different actions of insulin, resulting in improved metabolic and ovulatory functions [3]. Myo-inositol has been shown to improve oocyte energy status and quality, and DCI rapidly reduces the peripheral hyper insulinemia, hence found to be useful in the management of PCOS [4]. In recent years, several studies have proved the effectiveness of MI/DCI in patients with PCOS [5]. Myoinositol is necessary for metabolic management while DCI is equally important for menstrual, ovulatory, and cutaneous hyper androgenic resolution. The absolute concentrations of both inositols are important. Current evidence is inadequate to provide a definite answer regarding the optimal MI/DCI ratio. Formulations with relatively higher levels of DCI are usually preferred to circumvent epimerase deficiency and ensure adequate levels in the ovary [6]. Moreover, Indian data regarding the use of high concentration of DCI for PCOS treatment is scarce. Therefore, it is of interest to evaluate the treatment patterns, clinical effectiveness and safety of MI and DCI combination (550/150 mg) in management of PCOS among Indian women.

## Methods:

### Study characteristics:

This was a retrospective, multi-center, real-world study that collected data from 29 healthcare centers across India. The anonymized medical records from 283 women diagnosed with PCOS were accessed and data was entered into case report forms. The data collection for the study occurred between September 2019 and February 2020. Analyses of data were done as per the local laws and regulations and with due approvals from ethics committee (ACEAS Independent Ethics Committee, Ahmedabad: 20 Sep 2020).

### Study population:

Women aged 12-45 years diagnosed with PCOS, who had received myo-inositol 550 mg and D-chiro-inositol 150 mg tablets, were identified. The participants' treatment information was sourced from the treating physician under an agreement. Patients whose medical records contained a minimum core data set were included. The core data set included age, weight, blood pressure, androgen levels, hirsutism score, fasting plasma glucose (FPG), postprandial plasma glucose (PPG), serum lipid levels, insulin levels, homeostasis model assessment of insulin resistance (HOMA-IR). Ferriman-Gallwey scale for hirsutism was used for the measurement of hirsutism and the scoring was done by the treating physician.

### Intervention:

The treatment consisted of supplementation of one tablet of MI-DCI tablet twice daily for a period of three months. Each tablet contains combination of myo-inositol 550 mg and D-chiro-inositol 150 mg.

### Outcomes measures:

The main outcomes of the study included quantitative changes in weight, follicle-stimulating hormone (FSH), luteinizing hormone (LH), dehydro epiandrosterone (DHEA), hirsutism score, FPG and PPG, insulin levels, HOMA-insulin resistance (IR) index, and serum lipid profile. Various qualitative factors were evaluated including reduction of acne and acanthosis nigricans; restoration of the menstrual cycle; and spontaneous ovulation. Several population characteristics were also analyzed including the mean age of patients receiving MI-DCI tablets; duration of diabetes; and co-morbidities. Physicians were also asked to provide the final assessment of MI-DCI tablets efficacy and tolerability in each patient on a five-point scale: "fair", "average", "good", "very good" or "excellent". The ratings were based on the physicians’ assessment only, and no description of the categories was provided. Further, safety analysis was also performed. Where adverse events (AEs) were assessed and recorded by the physician.

### Statistical analysis:

The descriptive statistics for continuous variables were presented with the number (n) of observations, mean, and standard deviation (SD). For categorical data, descriptive statistics were presented with the number of exposed subjects and the percentage of observations in various categories. The continuous data were statistically analyzed and all p-values (95% Confidence Interval) were reported based on the paired t-test performed using GraphPad Prism 5.

## Results:

### Clinical characteristics:

A total of 283 patients with confirmed PCOS were included in this study. Table 1(see PDF) represents a demographic overview and characteristics of the study population. The mean age and body mass index of the patients was 27.74 years and 26.89 kg/m2 respectively. Sedentary lifestyle was the most prevalent risk factor (60.42%) in patients diagnosed with PCOS, followed by emotional stress (44.17%), family history of diabetes mellitus (30.04%) and PCOS (20.85%). The most commonly associated clinical abnormalities observed in patients with PCOS were oligomenorrhea (75.27%), infertility (65.02%), hirsutism (49.12%), and acne (42.76%). A total of 37 patients received metformin, 37 patients received letrozole, 10 patients received clomiphene citrate and 48 patients received oral contraceptive pills including cyproterone acetate, medroxy progesterone acetate, dydrogesterone, drospirenone, ethinyl estradiol, and levonorgestrel.

### Effect on hirsutism:

The data of pre-intervention of MI-DCI showed higher average hirsutism scores (n=142, 68.93%). While improvement in hirsutism score in the study population was observed after the intervention (n=97, 49.49%). Proportion of participants with moderate hirsutism reduced from 32.52% to 6.12%, while no participant was found to have severe hirsutism after Mychiro therapy (Figure 1-see PDF).

### Effect on serum lipid levels:

Patients who regularly consumed MI-DCI tablets exhibited statistically significant improvements in serum lipid levels (Figure 2A-D - see PDF). A statistically significant reduction was also observed in mean low-density lipoprotein-cholesterol [Difference: 9.81; 95% CI (6.68, 12.95); P<0.001] and total cholesterol [Difference: 19.89, 95% CI (13.14, 26.63); P<0.001] and triglyceride [Difference: 15.15; 95% CI (9.8, 20.5); P<0.001] levels after regular use of MI-DCI tablets. No significant effect was observed on the HDL-C levels. The mean LDL-C levels were found to be reduced by 9.81 mg/dL from the baseline levels of 106.40 mg/dL. TC and TG levels also considerably reduced by 19.89 mg/dL and 15.15 mg/dL from the baseline values of 210 and 144.18 mg/dL, respectively.

### Effect on LH/FSH ratio and Androgen levels:

Regular use of MI-DCI tablets resulted in a significant reduction in (LH/FSH ratio) and androstenedione levels (Figure 2E - see PDF). A decrease from baseline to post-intervention period in LH/FSH ratio was observed [Difference: 0.25; 95%CI (0.04, 0.47); P=0.021]. Concerning the mean blood androstenedione levels, a significant reduction was found from the baseline to end of the study [Difference: 0.18ng/mL; 95%CI (0.11, 0.24); P< 0.001] (Figure 2F - see PDF). A statistically significant reduction was also observed in mean free testosterone [Difference: 1.49pg/mL; 95% CI (0.79, 2.19; P<0.001] and dehydroepiandrosterone [Difference: 21.49 ng/mL, 95% CI (14.90, 28.07); P<0.001] levels after regular use of MI-DCI tablets (Figure 2G and H - see PDF).

### Effect on Insulin levels, Insulin resistance, and glycemic parameters:

The effect of MI-DCI treatment on serum insulin, insulin resistance, and glycemic parameters are depicted in Figure 2I to L(see PDF). A significant decrease in the mean serum insulin levels was observed from 85.01 to 75.11 mIU/L, P<0.001. A statistically significant reduction was also observed in FPG level [Difference: 8.20(mg/dL), 95% CI (5.37, 11.02); P<0.001], and PPG [Difference: 7.92(mg/dL), 95% CI (4.86, 10.97); P<0.001] levels after regular use of MI-DCI tablets. This corroborated with a decrease in insulin resistance index (HOMA-IR) by 0.29 (95% CI, 0.08-0.49,p<0.01) following treatment with MI-DCI.

### Clinical status and outcome:

After three months of intervention, remarkable reduction was observed in body weight in a wide majority of the patients (69.6%) (Table 2 - see PDF).

### Effect on Acne and Acanthosis Nigricans:

A remarkable improvement in acne was observed in 43.11% of patients with PCOS. Similarly, considerable improvement in Acanthosis Nigricans was observed in 31.1% of the participants, after 3 months of treatment with MI-DCI (Table 2 - see PDF).

### Adverse events:

Adverse events were reported in 4 patients which included nausea, diarrhea, headache, and vomiting (n=1, 0.35%; each, Table 2 - see PDF).

### Effect on menstrual cycle restoration and spontaneous ovulation:

The regular use of MI-DCI tablets was found to restore menstruation and spontaneous ovulation in 80.92% and 32.86% of the study population, respectively (Figure 3 - see PDF).

### Physician's Global evaluation of efficacy (PGEE) and tolerability (PGET):

Physicians rated the overall efficacy and tolerability of the MI-DCI tablets on a five-point scale. It was observed that the overall efficacy rating ranged between good and excellent, with physicians reporting excellent and very good efficacy in 13.9% and 47.8% of patients, respectively (Figure 4 - see PDF).The physicians' ratings suggested that the MI-DCI tablet has excellent and very good tolerability in 13.1% and 49.8% of the patients, respectively. For 31.7% of patients, physicians rated the tolerability as good (Figure 4 - see PDF).

## Discussion:

PCOS and obesity frequently co-occur where weight gain contributes to the development of PCOS [6] while the latter can aid further weight gain and make an effective weight loss more difficult. Their association also increases the challenge of their management as well as the risk of other morbidities such as cardio-metabolic dysfunction and insulin resistance [8]. Acne, hirsutism, and acanthosis nigricans are a few common dermatological manifestations of PCOS. Inositols have been shown to improve skin conditions via the reduction of hyper androgenism [9]. In our study, 43.11% and 31.1% of the participants reported remarkable improvements in acne and Acanthosis nigricans, respectively. Myo-inositol has been demonstrated to improve acne by reducing hyper androgenism in PCOS patients within 8 weeks along with improvements in hirsutism and menstrual irregularities [9]. Further, Ramanan *et al.* evaluated a combination of Myo-inositol with folic acid and vitamin D3, in resolving acne in women of menstruation age who are overweight and had normal hormone levels, and found that acne-related lesions were reduced as quickly as 8 weeks [9]. Similarly, a pilot study conducted in 15 patients with PCOS showed significant improvement in acne score after 3 months of therapy with myo-inositol, DCI and glucomannan [6].

Minozzi *et al.* evaluated the effects of Myo-inositol on hirsutism in 46 women and demonstrated a significant reduction in the hirsutism score from 13.1 (baseline) to 10.8 (6 months) (p<0.001) [10]. In the present study, significant improvement in hirsutism scores was observed among participating women after the regular consumption of MI-DCI tablets. Menstrual irregularity (oligo- or amenorrhea) is one of the defining features of PCOS [11]. Restoration of menstruation and spontaneous ovulation in our study suggests the positive impact of MI-DCI on the spontaneous ovulation activity. These results are in line with the previous studies that demonstrated the role of myoinositol in menstrual cycle restoration in women with PCOS [12,13].

Hyper androgenemia is one of the hallmarks of PCOS that is manifested as hirsutism, alopecia, or acne. An estimated 80% of women exhibiting these signs of hyper androgenism are diagnosed with PCOS [1]. The regular use of MI-DCI tablets in our study positively modulated the LH/FSH ratio, and blood levels of rostenedione, free testosterone, and DHEA. These results are in line with the previous observation of reduced testosterone levels with MI-DCI treatment in women with PCOS. Benelli *et al.* evaluated the effects of MI-DCI combination in young overweight women (N=46) with PCOS, and demonstrated a numerical reduction in rostenedione levels and a statistically significant reduction in free testosterone levels from 0.76 ng/mL at baseline to 0.62 ng/mL, p<0.05 [14]. There was remarkable reduction in LH, fasting insulin, and HOMA index in patients treated with MI-DCI combination therapy versus placebo [13]. In a previous study the Myo-inositol group had significantly reduced serum total testosterone from 99.5 to 34.8 ng/dL, and free testosterone from 0.85 to 0.24 ng/dL when compared to those in the placebo group, p=0.01) [13]. The LH/FSH ratio was attenuated after twice-daily treatment with MI-DCI combination therapy in a previous study (n=200) reporting reduced gonadotropin ratio from baseline (2.85) to 12 weeks (2.47) and 24 weeks (1.83) [15].

PCOS is accompanied by insulin resistance affecting 65-70% of all patients [1]. This impaired insulin sensitivity results in compensatory hyper insulinemia that may further enhance the stimulating effects of LH on androgen production in ovarian theca cells and contributes to androgen-dependent an ovulation via different mechanisms [1]. Reportedly, a defective insulin pathway can result from impairment in the inositol phosphoglycan (IPG) mediator that plays a role in activating the enzymes involved in glucose metabolism. The deficiency of inositol in IPG results in insulin resistance. Thus, in PCOS the appearance of insulin resistance could be due to altered metabolism of inositol or IPG mediators [16]. Significant reduction in the mean serum insulin levels demonstrated by the current study may suggest improved insulin sensitivity after treatment. Another study by Kachhawa et al evaluated the efficacy of MI-DCI, demontrated that MI-DCI is effective in regularizing menstrual cycles and improving insulin resistance [17]. This corroborated with a significant decrease in FPG, PPG, and insulin resistance index (HOMA IR) following treatment with MI-DCI. Our results are in line with the previous evidence that suggests the positive impact of inositol treatment in PCOS. In a previous study, MI supplementation for 12 weeks was found to significantly reduce the FPG (97.5 mg/dL to 89.8 mg/dL) and serum insulin levels (13.0 mIU/L to 10.8 mIU/L) than metformin [16].

Lipid abnormalities are common among women with PCOS with almost 70% of patients having altered lipid levels [18]. Dyslipidemia not only plays an important role in the development of PCOS but also increases the risk of cardiovascular disease in patients with PCOS [19]. In our study, almost 10% of the patients were reported to have dyslipidemia at baseline. The combination of MI and DCI has previously been shown to positively impact serum lipid levels in women with PCOS. In a previous study six months of treatment with combination therapy resulted in significant improvements in LDL-C (3.5 to 3.0, p<0.05) [10]. The present study showed significant improvements in lipid profile. These metabolic benefits are suggestive of probable cardiovascular risk reduction that can be achieved in women with PCOS, after regular treatment with MI-DCI tablets. Reportedly, dyslipidemia in PCOS is associated with insulin resistance, oxidative stress, hyper androgenism, and an ovulation. Thus, improvements in the lipid profile may exert multi factorial effects in PCOS. The remarkably low reports of adverse events in patients after regular consumption of MI-DCI tablets indicate its good tolerability. The high rating for efficacy and tolerability given by physicians also supports the efficacy and tolerability of MI-DCI tablets.

The present study was a retrospective evaluation; hence care must be taken when generalizing the results. Considering the real-world practice, the possibilities of bias in patient selection, and change in patient behavior could not be ruled out. Also, the physician's preferences, opinions, and decisions may vary in real-world practice. Despite these limitations such studies offer an opportunity to monitor the efficacy and tolerability of medication in routine clinical practice, which may more accurately reflect the real-world experiences of patients and physicians than can be achieved by controlled clinical trials. Thus, observational studies provide a valuable supplement to randomized controlled trials.

## Conclusion:

PCOS with its multidimensional implications predisposes affected women to various reproductive and metabolic disorders. MI-DCI helps in improving the hormonal, glycemic, and lipid profile of women with PCOS reducing the risk of various reproductive and cardio-metabolic diseases. Thus, MI-DCI, with its efficacy and tolerability is a promising choice in PCOS therapeutics.

## Contributors:

Dr. Abhijit Barua, Dr. Amarena Nath Das, Dr. AnghaPairaturkar, Dr. Archana Mayekar, Dr. Ashok Roy, Dr. Basab Saha, Dr. Bhanita Deka, Dr. Dipti Singh, Dr. Harpreet Kaur, Dr. Jaideep M Mehta, Dr. Karumanchi Nalini, Dr. Lila Vyas , Dr. Mangayarkarasi, Dr. MeetaAngadi, Dr. Padmaja Joshi, Dr. Poonam Goyal, Dr. Preetam Pal, Dr. Rajesh Soneji, Dr. ReetaHazra , Dr. Shashikala N, Dr. Shilpi Sud, Dr. Sonal Agrawal, Dr. Sujata Kelkar, Dr. Swetha Tumala, Dr. Tushar Zaveri, Dr. Umema Jamali, Dr. Veena Aggarwal, Dr. Venugopal M, Dr. Yazhini Selvaraj.

## Funding:

The study was funded by USV Pvt Ltd. Mumbai.
